# Dopamine-Independent Locomotor Actions of Amphetamines in a Novel Acute Mouse Model of Parkinson Disease

**DOI:** 10.1371/journal.pbio.0030271

**Published:** 2005-08-02

**Authors:** Tatyana D Sotnikova, Jean-Martin Beaulieu, Larry S Barak, William C Wetsel, Marc G Caron, Raul R Gainetdinov

**Affiliations:** **1**Department of Cell Biology, Center for Models of Human Disease, Institute for Genome Sciences and Policy, Duke University Medical Center, Durham, North Carolina, United States of America,; **2**Psychiatry and Behavioral Sciences, Mouse Behavioral and Neuroendocrine Analysis Core Facility, Duke University Medical Center, Durham, North Carolina, United States of America; University of Texas Southwestern Medical CenterUnited States of America

## Abstract

Brain dopamine is critically involved in movement control, and its deficiency is the primary cause of motor symptoms in Parkinson disease. Here we report development of an animal model of acute severe dopamine deficiency by using mice lacking the dopamine transporter. In the absence of transporter-mediated recycling mechanisms, dopamine levels become entirely dependent on de novo synthesis. Acute pharmacological inhibition of dopamine synthesis in these mice induces transient elimination of striatal dopamine accompanied by the development of a striking behavioral phenotype manifested as severe akinesia, rigidity, tremor, and ptosis. This phenotype can be reversed by administration of the dopamine precursor, L-DOPA, or by nonselective dopamine agonists. Surprisingly, several amphetamine derivatives were also effective in reversing these behavioral abnormalities in a dopamine-independent manner. Identification of dopamine transporter- and dopamine-independent locomotor actions of amphetamines suggests a novel paradigm in the search for prospective anti-Parkinsonian drugs.

## Abstract 2

Identification of dopamine transporter- and dopamine- independent locomotor actions of amphetamines suggests a novel paradigm in the search for prospective anti-Parkinsonian drugs.

## Introduction

The phenylethylamine derivative dopamine (DA) is critically involved in a wide variety of vital functions such as locomotion, feeding, emotion, and reward [1–3]. Major DA systems in the brain originate from brainstem DA neurons located in the substantia nigra pars compacta (SNc) and the ventral tegmental area (VTA). SNc neurons project mainly to the caudate/putamen or dorsal striatum (nigrostriatal system), whereas VTA neurons send their axons to the ventral striatum including the nucleus accumbens, as well as certain other limbic (mesolimbic system) and cortical areas (mesocortical system). Small DA-containing cell groups located primarily in the hypothalamus comprise the tuberoinfundibular DA system [4–6]. DA is synthesized from tyrosine by the rate-limiting enzyme tyrosine hydroxylase (TH), to produce L-DOPA which is quickly decarboxylated by *L*-aromatic acid decarboxylase (L-AADC) to DA [1,3]. Intraneuronal DA is accumulated into synaptic vesicles by the vesicular monoamine transporter-2 (VMAT2) [7,8]. DA released into the extracellular space exerts its physiological functions via activation of G protein-coupled D1-like and D2-like DA receptors [9]. Finally, DA in the extracellular space is subject to dilution by diffusion and metabolic degradation; however the major route of DA clearance from the extracellular space in the striatum/nucleus accumbens is the rapid recycling of the neurotransmitter back into dopaminergic terminals by the Na^+^/Cl^−^–dependent plasma membrane dopamine transporter (DAT) [10,11]. Recycled DA in the dopaminergic terminals is then stored in the large intracellullar storage pool available for subsequent re-release [12,13] .

It is well established that DA neurotransmission in both dorsal and ventral striatum is essential for normal locomotor functions, and progressive degeneration of DA neurons in these areas is a known cause of Parkinson disease (PD). In most cases, PD becomes clinically apparent when the loss of dopaminergic neurons reaches 60%–70%, which leads to functional dysregulation of the related neuronal circuitry [14–17]. Major motor manifestations of DA deficiency in PD include, but are not limited to, resting tremor (tremor occurring in the absence of voluntary movement), rigidity (tonically increased muscle tone), bradykinesia/akinesia (slowness/difficulty in initiating movement), gait disturbance and postural instability, facial masking, and decreased eye-blinking [18]. Presently, there is no known cure for PD [19,20], however its symptoms can be controlled by therapeutic interventions [21]. DA replacement therapy by administration of the DA precursor, L-DOPA, has been used for many years and remains the gold standard for treatment of PD [22,23]. However, the efficacy of this treatment wanes with time, and fluctuations in motor performance as well as psychotic reactions and dyskinesias often develop. DA agonists, as well as several other classes of drugs directly or indirectly affecting DA function (monoamine oxidase [MAO] inhibitors, COMT [catechol-o-methyl transferase] inhibitors, and amantadine), have some beneficial effects in PD patients, but they are mostly used either at early stages of PD or are applied as adjunct medications to enhance the benefits of L-DOPA [21,24,25]. Due to these limitations of existing therapeutic approaches, the development of better anti-Parkinsonian drugs remains a major objective of PD research. Several lines of evidence suggest that development of novel non-dopaminergic approaches aimed at bypassing impaired dopaminergic transmission would be beneficial in PD, particularly at later stages [16,26–28], however it is still unclear if these treatments would just potentiate action of residual DA or act completely independently of DA.

A number of animal models of DA deficiency, based on pharmacologic, neurotoxic, or genetic approaches, have been developed to understand basic pathological processes leading to PD and/or to search for novel principles of therapy [29–36]. However, in rodents, the prolonged absence of DA is not compatible with life [3,7,8], and animals with chronic severe DA depletion are generally not available for routine experimentation. We have developed mice lacking a functional DAT (DAT-KO mice) [11] that display remarkable alterations in the compartmentalization of DA [12,13,37]. Lack of the DAT-mediated inward transport in these mice results in an elevated extracellular DA and at least 95% decreased intracellular DA stores. Unlike normal animals, these mice demonstrate remarkable dependence of the remaining DA on ongoing synthesis, and pharmacologic blockade of DA synthesis in DAT-KO mice provides an effective approach to eliminate DA acutely [12,13].

Substituted phenylethylamine derivatives, amphetamines, that are structurally similar to DA and the endogenous trace amine β-phenylethylamine, represent a well-known group of compounds that potently affect psychomotor functions. Amphetamines are known to interact with plasma membrane monoamine transporters, including DAT, norepinephrine (NE) transporter (NET), and serotonin transporter. This complex interaction results in transporter-dependent efflux of monoamines into extracellular space from intraneuronal stores [10,38,39]. It is commonly believed that DAT-mediated efflux of DA is primarily responsible for the psychostimulant and hyperlocomotor actions of these drugs [38,40,41]. Intriguingly, recent studies have identified novel transporter-independent targets of amphetamines. It has been shown that amphetamines, as well as β-phenylethylamine, some monoamine metabolites, and several drugs affecting monoaminergic transmission, can directly activate specific G protein–coupled trace amine (trace amine 1 [TA1]) receptors [42] with currently unknown functional consequences [43,44].

We report here that the pharmacologic inhibition of the rate-limiting enzyme of DA synthesis, TH, almost immediately depletes brain DA to undetectable levels in DAT-KO mice and induces a transient recapitulation of essentially all PD symptoms for up to 16 h. DA-deficient DAT-KO mice (DDD mice) thus represent an acute PD model that is useful for studying the efficacy of compounds that potentially can restore control of locomotion in the absence of any contribution of the dopaminergic system. By using this approach, we found that several amphetamine derivatives can counteract the behavioral manifestations of severe DA deficiency, suggesting that, in addition to well-known DA-mediated effects, amphetamine-like compounds can also affect motor functions in a DA- and DAT-independent manner.

## Results

### A Pharmacologic Approach for Provoking Selective DA Deficiency in DAT-KO Mice

The ability of α-methyl-*p*-tyrosine (αMT), a potent irreversible inhibitor of TH [29,45,46], to impede production of brain DA suggests a simple, but straightforward, strategy for producing an acute PD mouse model. However, numerous studies have documented that treatment of normal animals with αMT results only in a relatively slow and partial depletion of DA in brain tissues that is not sufficient for generation of PD-like symptoms [29,45,46]. This limited depletion is based upon how DA is stored. It is believed that the large intraneuronal DA storage pool that normally exists in striatal DA terminals provides sufficient DA to release and recycle back into releasing terminals up to the time when newly synthesized TH starts to regain its functional role [29,45,46]. Thus, in a normal animal, complete depletion of striatal DA is unachievable by TH inhibition alone, and additional depletion of vesicular DA by VMAT2 inhibitors, such as reserpine is required [33,47–49]. Protocols designed for wild-type [WT] mice that use a dual inhibitor strategy (VMAT2 plus TH inhibitors) deplete DA to 1%–2% of control levels [33,47–50], but the levels of other monoamine neurotransmitters that are substrates for VMAT2 are also severely affected. This nonselective targeting of monoaminergic signaling generally results in very complicated phenotypes that are not necessarily reflective of classic PD.

In the absence of any pharmacologic treatment, the intraneuronal vesicular stores of DA in the striatum of DAT-KO mice are already profoundly depleted by at least 20-fold [12]. This selective depletion of DA in dopaminergic terminals of DAT-KO mice, as well as analogous depletion observed in mice lacking NET [51] or serotonin transporter [52] with NE and serotonin (5-HT), respectively, reflects the critical role of transporter-mediated recycling in the maintenance of intracellular storage pools [13]. With loss of the major intracellular storage pool of DA in DAT-KO mice, both the intracellular and extracellular levels of DA in the striatum become critically dependent upon ongoing DA synthesis. Therefore in DAT-KO mice, acute TH inhibition alone by αMT is sufficient to induce profound depletion of DA [12,13,37].

**Figure 1:**
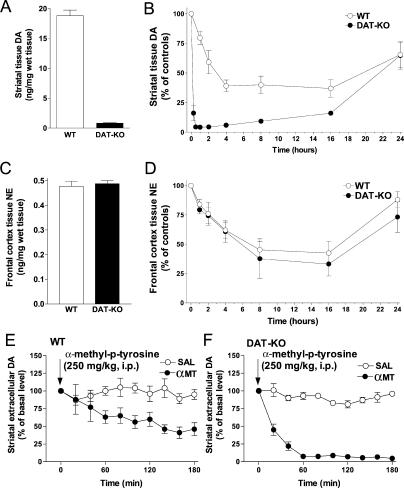
αMT Induces Severe DA Depletion in the Striatum of DAT-KO Mice (A) Tissue levels of DA in the striatum of saline-treated control WT and DAT-KO mice (*n* = 7 per group). Striatal levels of DA were significantly lower in DAT-KO versus WT mice (*p* < 0.05, Student's *t*-test). (B) Dynamics of the effect of αMT (250 mg/kg IP) on striatal tissue DA in WT and DAT-KO mice (*n* = 5–8 per group). DA levels were significantly lower versus control values at all the time points after αMT treatment in DAT-KO mice and 2–24 hours after treatment in WT mice (*p* < 0.05, one-way ANOVA followed by Dunnet's multiple comparison test). The magnitude of the effect was significantly different between genotypes from 1 to 16 h after αMT injection (*p* < 0.05, two-tailed Mann-Whitney *U* test). (C) Tissue levels of NE in the frontal cortex of saline-treated WT and DAT-KO mice (*n* = 7 per group). (D) Dynamics of the effect of αMT (250 mg/kg IP) on tissue levels of NE in the frontal cortex of WT and DAT-KO mice (*n* = 5–8 per group). NE levels were significantly lower versus control values at time points 2–16 after αMT treatment in DAT-KO mice and at 4–16 hours after treatment in WT mice (*p* < 0.05, one-way ANOVA followed by Dunnet's multiple comparison test). The magnitude of the effect was not different between genotypes at any time point after αMT injection (*p* > 0.05, two-tailed Mann-Whitney *U* test). (E) Effect of αMT on extracellular DA levels in the striatum of WT mice, measured using in vivo microdialysis. Data are presented as a percentage of the average level of DA measured in at least three samples collected before the drug administration. (Saline, *n* = 5; αMT, *n* = 7). αMT significantly decreased DA levels 60–180 min after treatment (*p* < 0.05, two-tailed Mann-Whitney *U* test versus respective time points in saline-treated controls). (F) Effect of αMT on extracellular levels of DA in the striatum of DAT-KO mice, measured by using in vivo microdialysis in freely moving mice. Data are presented as a percentage of the average level of DA measured in at least three samples collected before drug administration. (Saline, *n* = 4; αMT, *n* = 6). αMT significantly decreased DA levels 20–180 min after treatment (*p* < 0.05, two-tailed Mann-Whitney *U* test versus respective time points in saline-treated controls). Analysis of area under curve values for 120-min periods after drug administration revealed significant difference between DAT-KO and WT groups (*p* < 0.05, two-tailed Mann-Whitney *U* test). Note also that the basal extracellular levels of DA in DAT-KO mice were significantly higher than in WT mice (predrug concentrations of DA in dialysates were: WT, 76 ± 17 fmol/20 μl; DAT-KO, 340 ± 63 fmol/20 μl).

To explore this phenomenon in detail, we first measured the time-course of striatal DA depletion in DAT-KO and control mice following treatment with αMT (Figure 1). In agreement with previous studies [13], we observed that in saline-treated DAT-KO mice, striatal tissue levels of DA were about 20-fold lower than in WT controls (Figure 1A). The systemic administration of αMT (250 mg/kg IP) to DAT-KO mice produced rapid (15 min) and virtually complete (down to 5% of control levels in DAT-KO mice that is equivalent to less than 0.2% of WT control levels) depletion of striatal DA. In contrast, in WT mice the same treatment resulted in a relatively slow (4 h) depletion of only 60% of striatal tissue DA (Figure 1B). The duration of the depletion in DAT-KO mice was extensive, lasting up to 16 h, until a recovery of DA, related to the de novo synthesis of TH, occurs [29,45]. Notably, the rate of recovery of striatal DA levels was approximately the same in WT and DAT-KO mice.

Because DA itself serves as a precursor for neuronal production of NE in NE neurons, the inhibition of TH should also impact NE production. To test the impact of TH inhibition on the NE system, the frontal cortex tissue NE concentrations were measured in WT and DAT-KO mice. As opposed to the DAT, NET expression is not altered in DAT-KO mice so that the storage pool, which is by far the predominant reservoir of NE in NE-enriched regions such as the frontal cortex, should not be significantly altered in these mutants. Accordingly, the levels of NE in the frontal cortex tissue of saline-treated DAT-KO mice did not vary from that of WT mice (Figure 1C). Furthermore, αMT (250 mg/kg IP) treatment induced similar NE depletion in WT and DAT-KO mice by about 60% in 8 h after treatment. Importantly, the rates of partial NE depletion and recovery were almost identical between WT and DAT-KO mice (Figure 1D). Thus, TH inhibition in DAT-KO mice induces rapid severe depletion of DA, but only partially and slowly affects NE, indicating selectivity of this marked depletion to neurons expressing the DAT.

In order to demonstrate that targeting of TH by αMT depletes the functional extracellular pool of DA in living animals, we measured extracellular levels of striatal DA in freely moving mice by in vivo microdialysis. In agreement with total tissue DA data, αMT treatment essentially eliminated extracellular DA levels in DAT-KO mice (Figure 1F), whereas only a partial decrease was observed in WT mice. (Figure 1E). Thus, both intracellular and extracellular DA levels in the striatum of DAT-KO mice are critically dependent upon ongoing synthesis.

### DA Depletion in DAT-KO Mice Results in a Loss of Motor Control

**Figure 2:**
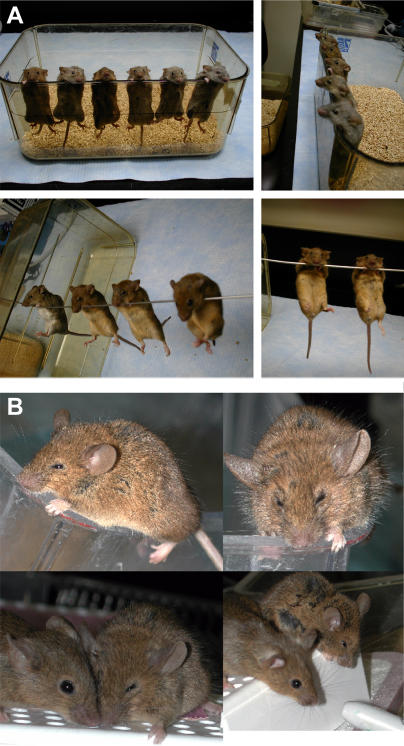
Display of Behavioral Phenotypes of DDD Mice (A) Akinesia and rigidity of DDD mice. Photos were taken 30 min after treatment of DAT-KO mice with αMT (250 mg/kg IP). (B) Ptosis in DDD mice. Photos were taken 3 h after treatment of DAT-KO mice with αMT (250 mg/kg IP). Lower panel: Mouse on the left side is saline-treated DAT-KO mouse, whereas the mouse on the right side is αMT-treated DAT-KO mouse.

**Figure 3:**
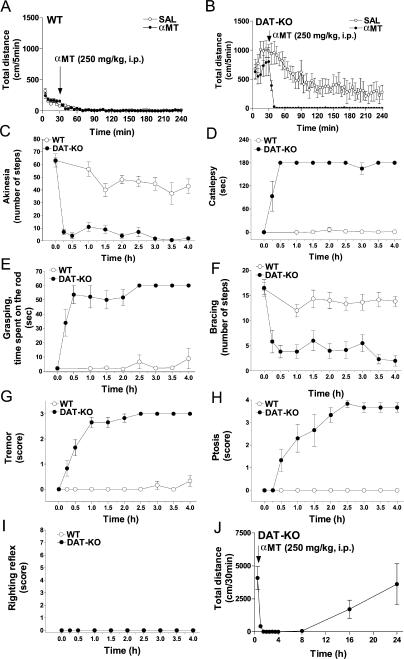
αMT-Induced Impairment in Motor Control in DAT-KO Mice Dynamics of locomotor activity following systemic administration of αMT (250 mg/kg IP) and saline (30 min after placement in the locomotor activity chamber) in WT (A) and DAT-KO (B) mice (*n* = 6–8 per group). Analysis of total distance traveled for 210 min after drug administration revealed significant effect of αMT treatment (*p* < 0.05; Student's *t*-test) in DAT-KO but not WT mice (WT-saline, 516 ± 50 cm/210 min; WT-αMT, 505 ± 98 cm/210 min; DAT-KO–saline, 18,489 ± 4,795 cm/210 min; DAT-KO–αMT, 448 ± 75 cm/210 min). αMT (injected at time 0) induced profound alterations in the akinesia (C), catalepsy (D), grasping (E), bracing (F) tremor (G), and ptosis (H) tests, but did not affect the righting reflex (I) in DAT-KO mice. Behavioral tests were performed as described in Materials and Methods. At all the time points, DAT-KO mice were significantly different versus respective values (data not shown) of saline-treated DAT-KO controls (*p* < 0.05; Student's *t*-test *n* = 6 per group) in these tests with exception of 15-min time point for ptosis (H) and all time points for righting reflex test (I). In WT mice only the akinesia test (C) revealed minor, yet significant, effect (1.5–4 h after αMT treatment) versus values (data not shown) of the respective saline treated WT controls (*p* < 0.05; Student's *t*-test; *n* = 6 per group). No significant alterations in any other test at any time point examined (D–I) was noted in αMT-treated versus saline treated (data not shown) WT mice. Locomotor activity is restored in DAT-KO mice 16–24h after αMT (250 mg/kg IP) treatment (J).

It is well known that DA plays a pivotal role in the control of various aspects of locomotor behaviors. Severe depletion of DA in αMT-treated DAT-KO mice results in a very specific akinetic phenotype (Figure 2; see Video S1). The DA-depleted DAT-KO mice (DDD mice) become akinetic almost immediately after treatment, in contrast to the essentially normal motor function displayed by αMT-treated WT mice. Moreover, DDD mice develop extreme rigidity, body tremor, and ptosis (droopy eyelids). These behaviors are evident on several tests (Figure 3). Akinesia was assessed by evaluating horizontal locomotor activity (Figure 3A and 3B) and by an “akinesia” test (Figure 3C); rigidity assessed by a catalepsy test (Figure 3D), a “grasping” test (Figure 3E), and a “bracing” test (Figure 3F); whereas tremor (Figure 3G) and ptosis (Figure 3H, see also Figure 2B) were visually determined [3,53–58]. These behaviors were analyzed in WT and DAT-KO mice for 4 h after a αMT treatment when depletion of DA is most severe in DAT-KO mice but with relatively minor effect on NE levels (see Figure 1). In all these measures DDD mice differed significantly from their WT littermates or saline-treated controls. Importantly, these abnormal behaviors in DDD mice, with the exception of ptosis, became maximal during the 30- to 60-min period following αMT exposure, thus correlating with the rate of DA depletion. Ptosis developed substantially later (Figure 3H), suggesting an additional contribution of NE depletion to the full magnitude of this response [59]. Importantly, the righting reflex of DDD mice was normal at all time periods analyzed (Figure 3I), indicating that this akinesia is not related to global sedation but rather to deficient movement control. It should be noted also that this global phenotype, which might be viewed as “freezing,” can be on some occasions temporarily disrupted by an acoustic startle or other stressful stimulus. However, after manifesting a few movements, the animals return to an akinetic state (data not shown). Strikingly, DDD mice, when placed in water, were able to swim with periods of floating and active swimming for at least a 3-min period (see Video S1), indicating that under certain conditions, movement can occur essentially without DA. Finally, in agreement with neurochemical data (see Figure 1B), the recovery from this profound akinetic phenotype in DDD mice occurs approximately 16–24 h following treatment (Figure 3J). The full recovery of animals allows repeated treatment with αMT, and, in fact, DAT-KO mice chronically treated with αMT (100 mg/kg, IP, once every 3 d) for a period of 40 wk showed no negative consequences [60].

### L-DOPA and Nonselective DA Agonists Restore Motor Activity in DDD Mice

**Figure 4:**
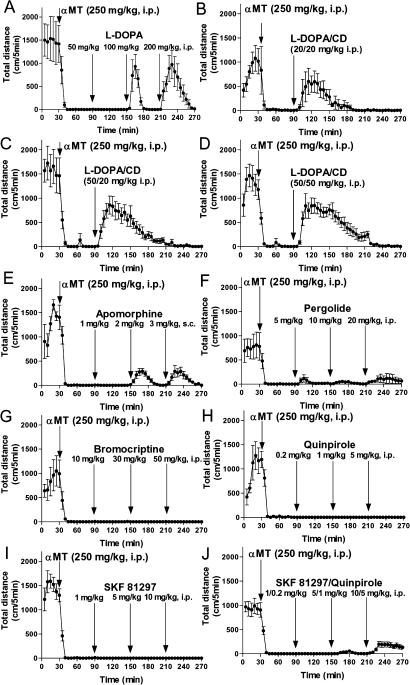
L-DOPA and Nonselective DA Agonists Are Effective in Restoring Locomotion in DDD Mice DAT-KO mice were placed in the locomotor activity chamber and 30 min later were treated with αMT (250 mg/kg IP) and 1 h after αMT were challenged with single or multiple doses of a drug (interval between treatments is 1 h). L-DOPA itself (A) or in combination with carbidopa (B–D) effectively restored locomotion in DDD mice, as revealed by the significant effect of L-DOPA at doses 100 and 200 mg/kg IP, or combinations of L-DOPA/carbidopa at doses 20/20, 50/20, and 50/50 mg/kg, IP (analysis of total distance traveled for 1 h after each dose of the drug; *p* < 0.05, two-tailed Mann-Whitney *U* test versus respective values in saline-treated DDD mice; data not shown). Nonselective DA receptor agonists, apomorphine (E) at doses 2 and 3 mg/kg SC, and pergolide (F) at doses 5, 10, and 20 mg/kg IP, induced locomotion in DDD mice (analysis of total distance traveled for 1 h after each dose of the drug; *p* < 0.05, two-tailed Mann-Whitney *U* test, versus respective values in saline-treated DDD mice; data not shown). D2 DA receptor agonists bromocriptine (G), quinpirole (H), and D1 DA receptor agonist (+)-SKF81297 (I) were not effective, but the combinations of D1 and D2 DA agonists (+)-SKF81297 plus quinpirole at doses 5/1 and 10/5 mg/kg IP, induced significant locomotion in DDD mice (analysis of total distance traveled for 1 h after each treatment; *p* < 0.05, two-tailed Mann-Whitney *U* test versus respective values in saline-treated DDD mice; data not shown). Experiments were performed in 6–12 mice per group.

The locomotor restoring effects exhibited by L-DOPA and DA agonists in various models of DA deficiency form one of the best-established paradigms in neuroscience [3,15,45,61]. As expected, high doses of L-DOPA alone (Figure 4A), or lower doses of L-DOPA given along with carbidopa (Figure 4B–4D) to reduce its peripheral metabolism via L-AADC inhibition, effectively restore locomotion in DDD mice. In fact, these treatments temporarily restore locomotion to the levels observed in untreated DAT-KO mice (Figure 4A–4D), which are normally at least 10 times more active than WT mice when placed into a novel environment [11,13]. Other manifestations associated with DA deficiency as described in Figure 3 were also essentially completely reversed (data not shown).

Efficacy of exogenous direct DA agonists was also tested in this model. Although the nonselective D1/D2 DA receptor agonists apomorphine and pergolide were somewhat effective in inducing forward locomotion (Figure 4E and 4F), the activity levels of DDD mice following these treatments were substantially lower than those induced by L-DOPA. Strikingly, the selective D1 DA receptor agonist (+)-SKF81297 and D2 DA receptor agonists, bromocriptine and quinpirole, were ineffective in inducing forward locomotion when administered separately (Figure 4G–4I). However, the combined administration of the D1 and D2 agonists (+)-SKF81297 plus quinpirole restored movement and induced forward locomotion (Figure 4J), supporting the well-established cooperative interaction of D1 and D2-like DA receptors in locomotor activity [62].

### Movement-Restoring Actions of Amphetamine Derivatives in DDD Mice

The loss of DA signaling that creates the motor symptoms of PD occurs upstream of many nondopaminergic pathways. This suggests that activation or inhibition of some of these downstream neuronal circuits could potentially reverse the motor deficits independent of restoration of DA activity. We, therefore, tested several non-dopaminergic compounds that potentially could reverse the consequences of severe DA deficiency in DDD mice (see Table 1). Many of these compounds have been found to be effective in restoring some aspects of movement control in one or another experimental animal model of PD and/or in PD patients [21,26,27,48,49]. However, in DDD mice none of the drugs were effective in restoring the major aspects of movement control required for forward locomotion (distance traveled). Although it is likely that the lack of locomotor effects of these drugs in DDD mice is related to an unprecedented level of DA depletion in these mice, it should be emphasized that in our studies only a few doses or combinations of drugs were tested. Furthermore, several treatments, although not inducing forward locomotion per se, were, nevertheless, somewhat effective in reversing other manifestations of DA deficiency. For example, the NMDA receptor antagonist MK-801 was able to reduce rigidity and promote weak, disorganized movement that however did not result in a significant increase in forward locomotion (Table 1). Synthetic amino acid L-DOPS (*L*-threo-3,4-dihydroxyphenylserine), which is decarboxylated to NE by L-AADC, selectively reversed ptosis in DDD mice. Cumulative dosing experiments revealed ptosis scores (measured 1h after each treatment) of 2.50 ± 0.28 after 100 mg/kg, 0 after 200 mg/kg, and 0 after 400 mg/kg IP of L-DOPS (*n* = 4), whereas corresponding values for saline-treated controls (*n* = 6) were 3.3 ± 0.3, 3.7 ± 0.2, and 3.7 ± 0.2, respectively. Effects of 200 and 400 mg/kg of L-DOPS on ptosis in DDD mice were significantly different as compared to respective control values (*p* < 0.05, Student's *t*-test) supporting an important role of NE in this behavioral manifestation [59]. Similarly, high doses of the trace amine β-phenylethylamine [44,63] (with or without concomitant inhibition of MAO) did not induce forward locomotion, but did promote weak stereotypic reactions, such as head-weaving and sniffing (data not shown). Further investigations will be required to fully evaluate the efficacy of these drugs in DDD mice.

**Table 1:**
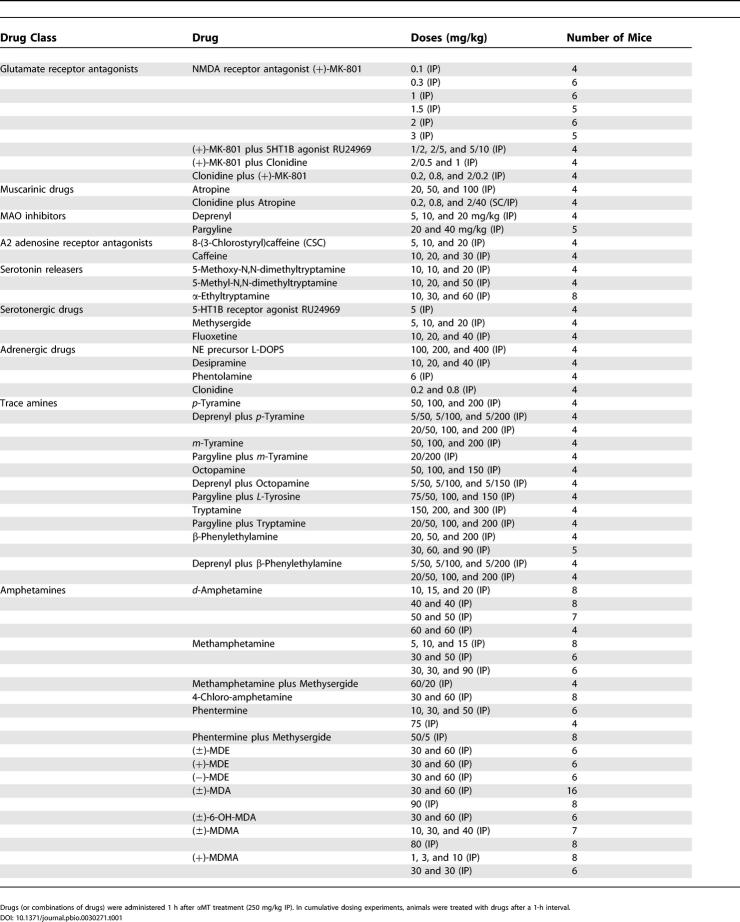
Treatments That Were Not Effective in Restoring Forward Locomotion in DDD Mice

**Figure 5:**
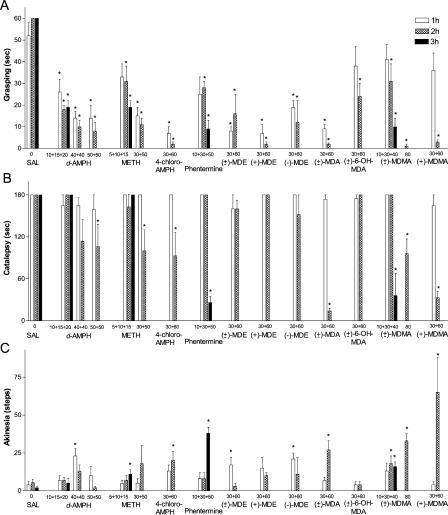
Amphetamine Derivatives at High Doses Are Effective in Reversing Abnormal Motor Behaviors of DDD Mice DAT-KO mice were placed in the locomotor activity chamber and 30 min later were treated with αMT (250 mg/kg IP), and 1 h after αMT were challenged with single or multiple doses of drugs (in cumulative dosing experiments, the interval between treatments was 1 h). Grasping (A), catalepsy (B), and akinesia (C) tests were performed as described in Materials and Methods 1 h after each dose (the only exception is (±)-MDMA at 80 mg/kg IP where measurements were performed 2 h after the drug administration). An asterisk indicates *p* < 0.05 versus respective values of saline-treated DDD mice (one-way ANOVA followed by Dunnet's multiple comparison test). Experiments were performed in 6–16 mice per group. *d*-AMPH indicates *d*-amphetamine; METH, *d*-methamphetamine; and 4-chloro-AMPH, 4-chloro-amphetamine.

Unexpectedly, this initial screening revealed a potent effect of amphetamine derivatives on behavioral manifestations of DDD mice. High doses of *d*-amphetamine, *d*-methamphetamine, 4-chloro-amphetamine, phentermine, (±)-MDE ((±)-N-ethyl-3,4-methylenedioxyamphetamine HCl), (+)-MDE ((+)-N-ethyl-3,4-methylenedioxyamphetamine HCl), (−)-MDE ((−)-N-ethyl-3,4-methylenedioxyamphetamine HCl), (±)-MDA ((±)-3,4-methylenedioxyamphetamine HCl), (±)-6-OH-MDA ((±)-6-hydroxy-3,4-methylenedioxyamphetamine HCl), (±)-MDMA ((±)-3,4-methylenedioxymethamphetamine HCl), and (+)-MDMA ((+)-3,4-methylenedioxymethamphetamine HCl) were effective in reducing manifestations of akinesia and rigidity in DDD mice as detected in the catalepsy, grasping, and akinesia tests (Figure 5A–5C). However none of these drugs (with the exception of (+)-MDMA, see below) was effective in restoring movement control sufficiently to induce forward locomotion (Table 1).

### DA-Independent Locomotor Effects of (+)-MDMA in DDD Mice

**Figure 6:**
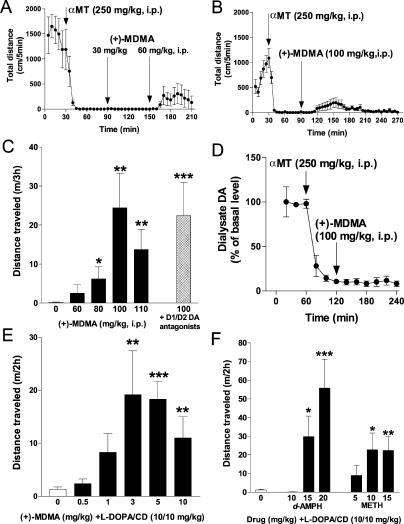
(+)-MDMA Induces Forward Locomotion in DDD Mice (A–C) DAT-KO mice were placed in the locomotor activity chamber and 30 min later were treated with αMT (250 mg/kg IP) and 1 h after αMT were challenged with single (B and C) or multiple doses (A) of a drug (interval between treatments is 1 h) (*n* = 10–16 per group). Repeated treatment with (+)-MDMA (30 and 60 mg/kg IP) induces forward locomotion in DDD mice (A). Analysis of total distance traveled for 1 h after 60 mg/kg IP of (+)-MDMA reveals significant effect of treatment versus respective period in saline-treated controls (p<0.05, two-tailed Mann-Whitney *U* test, data not shown). Dynamics (B) and dose-response (C) of locomotor effect of (+)-MDMA in DDD mice are shown. Pretreatment with D1 and D2 DA antagonists (SCH23390, 0.1 mg/kg SC plus raclopride, 2 mg/kg IP) 30 min before 100 mg/kg IP (+)-MDMA) did not affect locomotor action of (+)-MDMA (C). (D) (+)-MDMA (100 mg/kg IP) fails to affect DA dynamics in the striatum of DDD mice as measured by in vivo microdialysis. Data are presented as a percentage of the average level of DA measured in at least three samples collected before αMT administration (*n* = 4). Analysis of area under curve values for 120-min periods after (+)-MDMA administration revealed no significant difference in comparison with respective values in control group (Figure 1F; *p* > 0.05, two-tailed Mann-Whitney *U* test). (E and F) (+)-MDMA (E) as well as *d*-amphetamine and *d*-methamphetamine (F) at moderate doses potentiate locomotor-stimulating effect of subthreshold dose of L-DOPA/carbidopa (10/10 mg/kg IP). DAT-KO mice were treated with αMT as described above (A–C) and 45 min after αMT were injected with amphetamines. L-DOPA/carbidopa was injected 15 min after amphetamines, and distance traveled for 2h was measured (*n* = 6–15 per group). Note, that no forward locomotion was observed after these doses of (+)-MDMA, *d*-amphetamine and *d*-methamphetamine without L-DOPA/carbidopa, whereas L-DOPA/carbidopa (presented as drug dose 0) induced only a modest but significant (*p* < 0.05) increase in locomotion over saline-treated controls (data not shown). Single asterisk indicates *p* < 0.05; double asterisks indicate *p* < 0.01; and triple asterisks indicate *p* < 0.001 versus saline-treated controls (C) or L-DOPA/carbidopa-treated (10/10 mg/kg IP) group (E and F) (two-tailed Mann-Whitney *U* test). *d*-AMPH, *d*-amphetamine; METH, *d*-methamphetamine.

Among amphetamine derivatives, the most effective compound to counteract manifestations of akinesia and rigidity in DDD mice was (+)-MDMA (Figure 5A–5C). Thus, we tested (+)-MDMA in locomotor assay at even higher doses than those indicated in Table 1. As presented in Figure 6A–6C, (+)-MDMA at high doses was able to induce significant forward locomotion in DDD mice as measured by distance traveled in a locomotor activity test. This locomotor action of (+)-MDMA was observed in both cumulative (Figure 6A) and single dose (Figure 6B and 6C) treatments. In cumulative dosing experiments, a first treatment with 30 mg/kg of (+)-MDMA was not effective, but the subsequent administration of 60 mg/kg induced significant forward locomotion (Figure 6A) as well as reversal of other behavioral manifestations (see Figure 5A–5C) in DDD mice. Finally, testing of various single doses clearly showed a dose-dependence of the locomotor effect of (+)-MDMA in DDD mice (Figure 6C).

The locomotor stimulating effect of amphetamine and its derivatives are classically thought to result from the massive efflux of DA from presynaptic DA terminals via a mechanism including displacement of DA from vesicular storage and reversal of DAT-mediated DA transport [7,38–40]. However, in DDD mice, there is only a minimal amount of DA remaining (<0.2%) and the lack of the DAT precludes the possibility of amphetamine-mediated DA efflux. In fact, in vivo microdialysis studies confirmed that (+)-MDMA, at the effective dose necessary to induce significant locomotor activation in DDD mice, did not produce any detectable increase in striatal extracellular DA (Figure 6D). Moreover, this locomotor stimulation by (+)-MDMA was not inhibited by simultaneous blockade of D1/D2 DA receptors when DDD mice were pretreated with a combination of the D1 and D2 DA receptor antagonists, SCH23390 and raclopride (Figure 6C). Similarly, this pretreatment did not prevent the effects of amphetamine and phentermine on the akinesia and rigidity in DDD mice in grasping and akinesia tests (see Figure S1). In contrast, the same D1/D2 DA receptor blockade completely abolished the locomotor stimulating effects of L-DOPA/carbidopa (50/50 mg/kg IP) in DDD mice (see Figure S2). Taken together, these data indicate that (+)-MDMA can affect movement control in a DA-independent manner [64] and, most importantly, provide a proof-of-principle that pharmacologic activation of nondopaminergic neuronal pathways may be sufficient to restore movement even in the absence of DA neurotransmission.

It should be noted that the locomotor-stimulating effect of (+)-MDMA in DDD mice was observed only after high doses of the drug, which may be potentially neurotoxic [38]. However, the lack of the DAT renders dopaminergic neurons in DAT-KO mice significantly less sensitive to the neurotoxic effects of amphetamines, such as methamphetamine [65], as well as to MPTP (1-methyl-4-phenyl-1,2,3,6-tetrahydropyridine) [66,67]; thereby providing a unique opportunity to evaluate effects to large doses of amphetamines that would be impossible in normal animals [38]. It should be mentioned also that mice are generally less sensitive to MDMA neurotoxicity, particularly with regards to the serotonergic system [68]. Nevertheless, to directly evaluate the neurotoxic potential of MDMA in DAT-KO mice, we treated DAT-KO and WT mice with an established neurotoxic regimen of (±)-MDMA administration (4 injections of 20 mg/kg IP, every 2 h) [69] and assessed striatal tissue DA and 5-HT levels 7 d later. As might be expected, no significant differences in both DA and 5-HT levels were found between (±)-MDMA-treated and saline-treated DAT-KO mice (saline-treated DAT-KO mice (*n* = 6): DA, 0.53 ± 0.03 ng/mg tissue; 5-HT, 0.36 ± 0.03 ng/mg tissue; (±)-MDMA-treated DAT-KO mice (*n* = 7): DA, 0.58 ± 0.04 ng/mg tissue; 5-HT, 0.40 ± 0.02 ng/mg tissue), whereas the same regimen of treatment resulted in lethality of all treated WT mice (*n* = 7).

Furthermore, to test whether the locomotor-stimulating effect of (+)-MDMA may be evident under certain conditions with lower (nonneurotoxic) doses of the drug, we co-administered (+)-MDMA with a minimally effective dose of L-DOPA/carbidopa (10/10 mg/kg, IP.). As shown in Figure 6E, a potent synergistic effect of L-DOPA/carbidopa and (+)-MDMA was observed. Furthermore, similar effects were observed with relatively moderate doses of *d-*amphetamine and *d-*methamphetamine (Figure 6F). Thus, a DA-independent locomotor effect of amphetamines can be markedly enhanced with additional dopaminergic stimulation. It is also important to note that in a similar experiment, MAO inhibitor deprenyl (5, 10, or 20 mg/kg IP) failed to potentiate the effects of L-DOPA/carbidopa (data not shown), indicating that this effect is not related to the well-known MAO-inhibiting action of amphetamines [38].

### Nomifensine, but Not GBR12909 Affects Rigidity and Akinesia in DDD Mice

**Figure 7:**
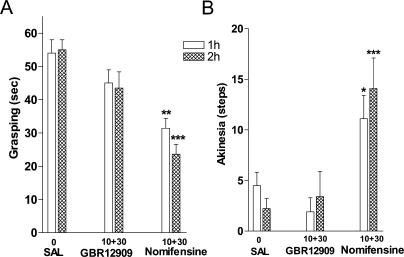
Nomifensine, but Not GBR12909, Is Effective in Reversing Abnormal Motor Behaviors of DDD Mice DAT-KO mice were placed in the locomotor activity chamber and 30 min later were treated with αMT (250 mg/kg IP). Mice were challenged 1 h later with two doses (10 and 30 mg/kg IP) of each drug or saline with a 1 h interval between treatments (*n* = 9–15 per group). Grasping (A) and akinesia (B) tests were performed as described in Materials and Methods 1 h after each dose. A single asterisk indicates *p* < 0.05, double asterisks indicate *p* < 0.01, and triple asterisks indicate *p* < 0.001 versus respective values of saline-treated DDD mice (one-way ANOVA followed by Dunnet's multiple comparison test). Note that no significant differences between GBR12909-treated mice and saline-treated controls in both tests were found, whereas nomifensine-treated mice were significantly different from GBR12909-treated mice (*p* < 0.05) in both experimental paradigms and doses tested.

Finally, to evaluate the potential of other TA1 receptor ligands for their ability to affect motor control in DDD mice, we elected to compare the effects of two potent DAT blockers that have been shown to be markedly different with regards to their activity at TA1 receptor. It has been recently reported that the mixed DAT and NET inhibitor nomifensine can also potently activate TA1 receptor whereas the selective DAT blocker GBR12909 completely lacks the ability to interact with TA1 receptor [42]. In DDD mice, both nomifensine and GBR12909 at doses tested (cumulative treatment with 10 and 30 mg/kg IP) were not effective in inducing forward locomotion or reversing catalepsy (data not shown). Nevertheless, nomifensine significantly reduced akinesia and rigidity in grasping and akinesia tests (Figure 7A and 7B), whereas no such effects were observed with equivalent doses of GBR12909 (Figure 7A and 7B).

## Discussion

In this study we demonstrate that inhibition of DA synthesis in DAT-KO mice represents a straightforward approach for developing an acute model of severe DA deficiency exhibiting a characteristic behavioral phenotype that can be utilized for testing perspective anti-PD treatments. Furthermore, these observations provide functional evidence for an important role of DAT-mediated recycling mechanism in the maintenance of intraneuronal DA. Finally, the novel DAT- and DA-independent locomotor action of amphetamines identified in these mice directly demonstrates the possibility of movement in a DA-independent manner.

### Role of DAT-Mediated DA Recycling in the Maintenance of Intraneuronal DA Storage

DAT is commonly known as a major regulator of the duration and intensity of extracellular DA signaling. However the important role of DAT in the control and maintenance of the intraneuronal DA storage pool frequently remains overlooked. It is generally assumed that the intraneuronal storage of DA is replenished primarily from newly synthesized DA with some contribution from recycled DA. However, several lines of evidence support a predominant role of DAT-mediated recycling of DA for the maintenance of the large storage pool in DA terminals. First, mice lacking the DAT display dramatically decreased (20-fold) striatal tissue DA content, that mostly represents intraneuronal DA concentrations. Second, as we demonstrate in the present study, the remaining DA in all compartments is extremely sensitive to TH inhibition. Furthermore, pharmacologic studies have shown that significant DA depletion may occur after administration of DAT inhibitors, particularly after chronic drug treatment [13]. Importantly, in the frontal cortex, where DAT levels are normally low in comparison to the striatum, tissue DA concentration is also low and can be more significantly affected than in the striatum by αMT [70]. It is likely that the newly synthesized DA does not contribute directly to the large storage pool of DA in nigrostriatal terminals, but rather contributes to it indirectly via released and recycled DA. Thus, a cooperative function of both DA synthesis and transporter-mediated recycling processes is necessary for the maintenance of normal presynaptic monoamine concentrations.

### A Novel Acute Mouse Model of Severe DA Deficiency, DA-Depleted DAT-KO (DDD) Mice

By using a combination of genetic and pharmacologic approaches we have developed a novel acute mouse model of severe DA deficiency, DDD mice. The lack of an active recycling mechanism in DAT-KO mice results in a profound depletion of intraneuronal concentrations of DA leaving the remaining DA entirely dependent on ongoing synthesis. As a result, inhibition of DA synthesis essentially eliminates striatal DA in these mice leading to the extreme behavioral manifestations. In fact, DDD mice demonstrate a unique set of behaviors that reproduces symptoms of PD with high fidelity. Thus, the lack of DA combined with the striking and highly reproducible behavioral phenotype in these mice can be used as an excellent tool to evaluate the potential of drugs that can affect locomotion in a DA-independent manner. Furthermore, by adapting the dose of αMT to produce various degrees of DA depletion, these mice can also be employed to find novel approaches to restore movement under conditions of partially impaired DA transmission that might be more relevant to most PD cases.

Several rodent models have been developed to understand pathological processes leading to PD and/or to screen for novel therapeutic strategies [29,30,34−36,71]. These models either recapitulate the loss of DA through pharmacologic or genetic manipulation, or recapitulate the neurodegenerative process through administration of selective neurotoxins and, recently, through mutations of specific proteins. However, in many of these models only incomplete and highly variable levels of DA depletion are achieved often precluding an accurate recapitulation of the neurological manifestations of PD. This poor behavioral expression of PD-related behaviors generally results in high level of false-positive results in drug screening tests in general, and particularly in those attempted to identify non-DA therapies [72].

Among several genetic mouse models of DA deficiency available today [73,74], the most effective was developed by inactivation of TH in DA neurons (DA-deficient [DD mice]) [3,75–81]. DD mice have provided important insights into the role of DA in movement control, feeding, and reward. This mutation results in severely impaired movement and feeding, which become apparent at 10 d and leads to death by 30 d. To maintain viable mice with the ability to move and feed requires daily treatment with L-DOPA, which results in an oscillation of striatal DA from about 1% to 10% over 24 h [77,81]. Many behavioral manifestations observed in DDD mice in this study, such as rigidity and akinesia, were observed previously in DD mice [3,76,79]. Importantly, both of these models showed temporal locomotor reactivity to stress and demonstrated normal righting reflex and ability to swim, indicating that certain movements may occur in a DA-independent manner. Despite these similarities, some important differences were noted between these two genetic models of severe DA dysfunction. In DD mutant mice, a lack of TH resulting in permanently decreased DA signaling, as well as daily treatments with L-DOPA render these mice extremely supersensitive to DA stimulations [81], whereas excessive DA signaling in DAT-KO mice results in compensatory down-regulation (but nonuniform) of DA receptors [11,13]. This may explain why certain behavioral manifestations of DA deficiency such as rigidity and akinesia may be more robust in DDD mice, whereas tremor was not observed in DD mutants [3,76]. Furthermore, efficacy of L-DOPA and DA agonists are remarkably higher in DD in comparison to DDD mice [3,76,81]. Additionally, several other drugs, such as caffeine and N-methyl-D-aspartate receptor antagonist MK-801, that are able to induce locomotion in DD mutants [75,80] are not effective in DDD mice (Table 1). In fact, down-regulation of DA receptor responsiveness combined with the extreme level of DA depletion in DDD mice may favor these mice as a very conservative approach for evaluating drugs that can affect locomotion in a DA-independent manner. Furthermore, rapid and effective elimination of DA in DDD mice may provide a simple in vivo approach to study DA receptor signaling [82] and/or to define neuronal circuitry involved in locomotor control [83].

### DA-Independent Locomotor Action of Amphetamines

Intriguingly, in both DD and DDD mice *d*-amphetamine was effective in restoring at least some aspects of locomotor behaviors. In DD mice, *d*-amphetamine (5 mg/kg IP) induced potent locomotor activation essentially up to the levels observed in WT controls. At the same time, a second treatment 2 h later by the same dose of the drug failed to induce locomotion in DD mice suggesting that this effect is dependent upon residual (after L-DOPA administration) DA which might be depleted by the first treatment with the drug [76]. In DDD mice, *d*-amphetamine itself was not able to induce forward locomotion at doses up to 60 mg/kg, but it produced significant effects on other manifestations of DA deficiency. Moreover, co-administration of relatively moderate doses of amphetamine (15 and 20 mg/kg) with a subthreshold dose of L-DOPA resulted in a marked locomotor activation of DDD mice. Thus, some DA tone seems to be necessary to express the full magnitude of locomotor activation by amphetamine, but it is evident that there is a DA-independent component of action that contributes to the overall effect of the drug. Further evidence for this idea relates to the fact that many other amphetamine derivatives are also active in reversing certain behavioral manifestations in DDD mice. Strikingly, both single and repeated treatment with (+)-MDMA was effective in inducing forward locomotion essentially without any contribution of DA. It is important to note that a potent anticataleptic effect of MDMA in haloperidol-treated rats [84] and antiakinetic effects in 6-OH-DA–lesioned rats [85] and MPTP-treated monkeys [64] have been recently reported. The present observations support these findings and suggest that these actions are not unique to MDMA but may be extended to other amphetamines. Further characterization of these unexpected effects of amphetamines may provide a novel framework in the search for potential anti-Parkinsonian drugs.

Amphetamine derivatives are known mainly as indirect enhancers of monoaminergic (DA, NE, and 5-HT) transmission via complex interactions with the plasma membrane monoamine transporters and the vesicular storage of these monoamines [7,10,12,38,39]. It should be reiterated that a lack of DAT in DAT-KO mice excludes the possibility of major effects of amphetamines on DAT-mediated DA efflux from presynaptic DA stores [40]. Furthermore, a blockade of D1/D2 DA receptors was ineffective in preventing the locomotor stimulating action of (+)-MDMA. Thus, it is virtually impossible that the observed effects of MDMA and other amphetamines in DDD mice are directly related to DA transmission. Although it is possible that this effect may be due to transporter-mediated action of amphetamines on NE or 5-HT transmission [38,40,86], it should be noted that among several NE- and 5-HT–related drugs tested (desipramine, clonidine, the NE precursor DOPS, fluoxetine, 5-methoxy-N,N-dimethyltryptamine, 5-methyl-N,N-dimethyltryptamine, α-ethyltryptamine, and 5-HT1B agonist RU24969), none were effective in DDD mice in tests of forward locomotion or akinesia and rigidity (data not shown). Similarly, no locomotor effect of MAO-A or MAO-B inhibitors was observed in these mice, indicating that the locomotor effect of amphetamines may not be explained by MAO-inhibitory action [38]. Furthermore, it should be underlined that locomotor actions of amphetamines observed in DDD mice occur at doses that are much higher than necessary to induce classic transporter-mediated effects [10,38,83].

Amphetamines share close structural similarity with an endogenous trace amine of unknown function β-phenylethylamine [87]. Amphetamines and β-phenylethylamine similarly interact with the plasma membrane monoamine transporters to elevate extracellular monoamine concentrations [63]. Intriguingly, recent evidence indicates that many amphetamine derivatives, including MDMA, may also act directly as agonists of trace amine TA1 receptors, that are known to be activated by β-phenylethylamine [42,88]. Several members of the family of trace amine receptors have been identified, however little is known about the pharmacology and functional role of these receptors in mammalian physiology [43,44,63]. It is reasonable to suggest that activation of TA1 receptors [42] or other trace amine receptors may provide a potential mechanism for DA-independent locomotor effect of MDMA and amphetamines in DDD mice. In line with this hypothesis, we observed that the DAT blocker nomifensine that can activate TA1 receptor, but not GBR12909 which is devoid this activity [42], is able to affect motor control in DDD mice. It should be noted, however, that in our initial exploration in DDD mice, we did not observe clear locomotor effects for any trace amine tested; but only a few doses, routes of administration, and combinations with enzyme inhibitors were investigated. Further detailed investigations will be needed to clarify the mechanism of locomotor action of amphetamines in DDD mice.

### Conclusions

In summary, these results provide additional functional evidence for the critical role of DAT in the maintenance of DA storage in presynaptic terminals. Rapid and effective abolishment of DA by inhibition of DA synthesis in DAT-KO mice provides a novel approach to develop severe DA deficiency that might be used to identify neuronal mechanisms involved in motor control in the absence of DA. Amphetamines are capable of affecting neuronal systems involved in motor control through mechanisms independent of DAT, in particular, and DA in general.

## Materials and Methods

### 

#### Animals

DAT-KO mice were generated as previously described [11]. Animal care was in accordance with the Guide for Care and Use of Laboratory Animals (National Institutes of Health publication #865–23, Bethesda, Maryland, United States) with an approved protocol from the Duke University Institutional Animal Care and Use Committee. C57BL/6J × 129Sv/J hybrid WT and DAT-KO mice, 3–5 mo old, of both sexes were used. None of animals used in these studies had the neurodegenerative phenotype sporadically observed in DAT-KO mice [60].

#### Drugs

Drugs or saline (0.9% NaCl) were administered intraperitoneally (IP) or subcutaneously (SC) in a volume of 10 ml/kg. The drugs were either from Sigma (St. Louis, Missouri, United States) or supplied by the National Institute of Drug Abuse (NIDA). Drugs provided by the NIDA Drug Supply Program included: (±)-MDMA, (+)-MDMA, (±)-6-OH-MDA, (±)-MDA, (±)-MDE, (+)-MDE, (−)-MDE, and AET (α-ethyl-tryptamine acetate).

#### Neurochemical assessments

Striatal tissue contents of DA and frontal cortical tissue levels of NE were assessed using HPLC-EC (high performance liquid chromatography with electrochemical detection) as described [8]. In vivo microdialysis measurements of striatal extracellular DA levels in freely moving mice were performed at least 24 h after implantation of a microdialysis probe as described previously [50]. Dialysate samples were assayed for DA using HPLC-EC.

#### Behavioral methods

Locomotor activity of littermate WT and DAT-KO mice was measured in an Omnitech CCDigiscan (Accuscan Instruments, Columbus, Ohio United States) activity monitor under bright illumination [83]. All behavioral experiments were performed between 10:00 AM and 5:00 PM. Activity was measured at 5-min intervals. To evaluate the effects of drugs on motor behaviors, mice were placed into activity monitor chambers (20 × 20 cm) for 30 min and then treated with αMT (250 mg/kg IP). A drug or combination of drugs were injected 1 h after αMT administration, and various parameters of locomotor activity were monitored for up to 3 h. In cumulative dosing experiments, animals were treated with increasing doses of drugs after a 1-h interval.

For the akinesia test, the mouse is held by the tail so that it is standing on forelimbs only and moving on its own. The number of steps taken with both forelimbs was recorded during a 30-s trial [57]. The presence of catalepsy was determined and measured by placing the animal's forepaws on a horizontal wooden bar (0.7 cm in diameter), 4 cm above the tabletop. The time until the mouse removed both forepaws from the bar was recorded, with a maximum cut-off time of 3 min [53]. In the grasping test of muscular rigidity, the mouse is suspended by its forelimbs on a metal rod (diameter: 0.25 cm) positioned approximately 20 cm above the table. The time the animal remains on the rod (maximum 1 min) was noted [58]. To assess rigidity in a bracing task, the number of steps taken with each forelimb when the mouse is pushed sideways over a distance of 50 cm was recorded [57]. Tremor was scored visually in mice using the rating scale [54]: 0, no tremor; 1, occasional isolated twitches; 2, moderate or intermittent tremor associated with short periods of calm; and 3, pronounced continuous tremor. Ptosis was scored as described [89]: 4, eyes completely closed; 2, half-open eyes; and 0, wide-open eyes; with 1 and 3 indicating intermediate values. The righting reflex was evaluated by turning the mouse onto its back five times. Normal mice immediately turn themselves over, to right themselves onto all four feet. Righting reflex was scored as follows: 0, no impairment; 1, on side one to two times; 2, on side three to four times; 3, on side five times; 4, on back one to two times; 5, on back three to four times; 6, on back five times; 7, sluggish when placed on back; and 8, righting response absent when on back and tail pinched [55].

#### Data analysis

The data are presented as mean ± SEM and analyzed using a two-tailed Student's *t*-test and one-way analysis of variance (ANOVA) followed by Dunnet's multiple comparison test or a two-tailed Mann-Whitney *U* test when appropriate.

## Supporting Information

Figure S1 D1/D2 DA Receptor Blockade Does Not Prevent the Effects of Amphetamine and Phentermine on Rigidity and Akinesia in DDD Mice (4.8 MB TIF).

Figure S2 D1/D2 DA Receptor Blockade Prevents the Locomotor Stimulating Effect of L-DOPA/Carbidopa in DDD Mice (4.4 MB TIF).

Video S1 Behavioral Phenotype of DDD mice (10 MB MOV).

## Abbreviations

5-HT: serotonin
αMT: α-methyl-p-tyrosine
DA: dopamine
DAT: dopamine transporter
DAT-KO mice: dopamine transporter knockout mice
DD mice: dopamine-deficient mice
DDD mice: dopamine-deficient DAT-KO mice
IP: intraperitoneal
L-AADC: *L*-aromatic acid decarboxylase
MAO: monoamine oxidase
NE: norepinephrine
NET: norepinephrine transporter
PD: Parkinson disease
SC: subcutaneous
SNc: Substantia Nigra Pars Compacta
TA1 receptor: trace amine 1 receptor
TH: tyrosine hydroxylase
VMAT2: vesicular monoamine transporter-2
VTA: ventral tegmental area
WT: wild-type
